# TIMP-1 Promotes Accumulation of Cancer Associated Fibroblasts and Cancer Progression

**DOI:** 10.1371/journal.pone.0077366

**Published:** 2013-10-15

**Authors:** Yixuan Gong, Evita Scott, Rong Lu, Yin Xu, William K. Oh, Qin Yu

**Affiliations:** 1 Department of Oncological Sciences, Department of Medicine, Tisch Cancer Institute, Icahn School of Medicine at Mount Sinai, Mount Sinai, New York, United States of America; 2 Division of Hematology/Medical Oncology, Department of Medicine, Tisch Cancer Institute, Icahn School of Medicine at Mount Sinai, Mount Sinai, New York, United States of America; University of Kentucky College of Medicine, United States of America

## Abstract

Treatment options for late stage prostate and colon cancer are limited and there is an urgent need to develop more effective and targeted novel therapies, which starts with identification and validation of novel therapeutic targets. Recent clinical studies have demonstrated that tissue inhibitor matrix metalloproteinase-1 (TIMP-1) levels are elevated in cancer patient plasma and elevated TIMP-1 levels are associated with worse clinical outcomes. However, it is unknown whether TIMP-1 serves merely as a biomarker of cancer progression or has a functional role in promoting cancer progression and can serve as a cancer therapeutic target, which is the main objective of this study. Here, we show that stroma of human prostate and colon cancer express higher levels of TIMP-1 compared to their normal counterparts and increased expression of TIMP-1 promotes *in vivo* growth of both cancer types. We demonstrate for the first time that increased TIMP-1 expression stimulates accumulation of cancer associated fibroblasts (CAFs) within prostate and colon cancer tissues and that TIMP-1 enhances prostate CAF proliferation and migration *in vitro* and promotes ERK1/2 kinase activation in these CAF cells. Our results establish the novel promotive effects of TIMP-1 on cancer progression and on accumulation of CAFs that in turn provides a pro-tumor microenvironment. Together, these results establish the potential of TIMP-1 as a novel target for cancer therapy and the mechanism underlying the pro-tumor activity of TIMP-1.

## Introduction

Prognosis for metastatic castration-resistant prostate cancer (CRPC) remains poor with a median survival of less than 2 years. Treatment options for the late stage prostate cancer are limited and there is an urgent need to develop more effective and targeted novel therapies [1-5]. During cancer progression, it is essential that cancer cells properly interact with and successfully modify their surrounding host microenvironment. Cancer cells interact with their microenvironment through the cell-surface adhesion receptors and the receptors for the extracellular matrix (ECM) and modify their surroundings largely through activities of the matrix metalloproteinases (MMPs). MMPs play key roles in degrading the ECM and bioactivities of MMPs are regulated by the tissue inhibitors of metalloproteinases (TIMPs) [6]. There are four members of the TIMP family, tissue inhibitor matrix metalloproteinase-1 (TIMP-1), -2, -3, and -4. TIMPs can inhibit the activities of all MMPs but with varying efficiency towards different MMPs. MMPs play important roles in tumor dissemination and progression and they are established therapeutic targets for a variety of cancer types [6-8]. Earlier studies indicated that TIMPs including TIMP-1 display anti-cancer activities [9-13]; however, recent studies have demonstrated a paradoxical pro-tumor effect of TIMP-1 [6,14-16]. TIMPs can affect cancer progression in a MMP-dependent and MMP-independent manner, though the underlying mechanism of the latter is not well understood [17,18]. 

Many broad-spectrum MMP inhibitors (MMPIs) developed by pharmaceutical companies were tested in prospective clinical trials and the results have been uniformly disappointing. No definitive mechanistic understanding for such failure has been established. However, it is likely resulted from a lack of understanding of the distinct bioactivities and functions of different MMPs, which have since been appreciated better for their pro- and/or anti-tumor activities [6,19-22]. These disappointing outcomes also emphasize the importance of better understanding of the biology of cancer therapeutic targets before conducting clinical trials. Recently, several clinical studies have demonstrated that TIMP-1 levels in cancer patient plasma and cancer tissues are highly elevated and the elevated TIMP-1 levels are associated with worse clinical outcomes in many cancer types including prostate and colon cancer [23-34]. However, it is unclear whether TIMP-1 serves merely as a biomarker of cancer progression or functions to promote cancer progression as well; and thus could serve as an important cancer therapeutic target. This question is addressed by this study. 

Here we show that stroma of human prostate and colon cancer express higher levels of TIMP-1 compared to their normal counterparts and that increased expression of TIMP-1 promotes *in vivo* growth of both cancer types. In addition, we demonstrate that TIMP-1 enhances prostate CAF proliferation and migration *in vitro* whereas knockdown of TIMP-1 inhibits prostate CAF proliferation and migration. In addition, TIMP-1 promotes activation of ERK1/2 kinase in these CAFs. We also show that increased expression of TIMP-1 stimulates accumulation of cancer associated fibroblasts (CAFs) within prostate/colon cancer tissues. Together, our results establish the novel promotive effects of TIMP-1 on the cancer progression and CAF accumulation, the potential of TIMP-1 as a novel cancer therapeutic target, and the mechanism underlying the TIMP-1 effects. 

## Materials and Methods

### Patient Cancer Samples, Cells, and Reagents

Human prostate and colon cancer samples were obtained from the Cooperative Human Tissue Network (CHTN) at the University of Pennsylvania and the Ohio State University. Primary prostate cancer associated fibroblasts (PCAFs) were obtained from the Asterand USA (Detroit) and cultured according to the manufacturer’s instruction. Human prostate fibroblasts were from the ScienCell (HPrF, Carlsbad) and cultured in the Fibroblast Medium (FM, ScienCell). PC3, PC3/M, DU145, 22RV1, LNCaP, VCaP, RWPE-1 and WPE1-NB26-65 human prostate cancer cells and HCT116 and HT-29 human colon cancer cells were from the NCI (DTP, DCTD Tumor Repository) and ATCC (Manassas) and maintained following the providers’ recommendations. Human prostate cancer LAPC-4 cells were obtained from the ATCC Patent Depository. 

Anti-v5 epitope (Invitrogen), -TIMP-1, -alpha smooth muscle actin (R&D Systems, Santa Cruz), -ERK1/2, -phospho-ERK1/2, -AKT, and -phospho-AKT (Santa Cruz, Cell Signaling), and CD63 (EMD Millipore) antibodies, and the Cell Proliferation Reagent WST-1 (TaKaRa) were used in the experiments. Purified human TIMP-1 was obtained from R & D Systems. 

### Reverse Transcriptase-Polymerase Chain Reaction (RT-PCR), Expression Constructs, and Virus Transduction

Full-length TIMP-1 cDNAs were generated by PCR and cloned along with their COOH-terminal v5-epitope tags to the retroviral expression vector pQCXIP (BD Biosciences) as described [35,36]. All expression constructs were verified by DNA sequencing. Retroviruses were generated using these expression constructs and pVSVG in GP2-293 cells following the manufacturer’s instructions (BD Biosciences). 

Human prostate cancer cells (PC3, 22RV1, and LAPC-4) and human colon cancer cells (HCT116 and HT-29) were transduced with the retroviruses carrying empty retroviral expression vector (as controls), or TIMP-1. Infected cells were selected for their resistance to puromycin and pooled populations of the drug resistant prostate and colon cancer cells with or without expression of exogenous v5-epitope tagged TIMP-1 were used in the experiments. Anti-v5 mAb (Invitrogen) was used to detect expression of exogenous TIMP-1 (TIMP-1v5). 

### Real-Time Quantitative PCR (qPCR)

Total RNA was isolated using the Trizol reagent (Life technologies, Carlsbad,) and used to synthesize cDNA with Superscript III platinum one-step qPCR kit (Life Technologies). QPCR analyses of human TIMP-1 mRNA expression were performed with Taqman gene expression assay (Hs00171558_m1) on ViiA7 real-time PCR machine with Taqman universal 2× master mix (Life Technologies). The gene expression levels were normalized to endogenous actin control (primer-limited probe, Life Technologies). 

### ELISA Analysis

TIMP-1 in prostate cancer cell culture supernatants and patient sera were measured by human TIMP-1 ELISA Duoset (R&D Systems) according to the manufacturer’s instructions. Briefly, for measurement of TIMP-1 in cell culture supernatants, 2×10^6^ cells were seeded into 60 mm dishes. After all cells have attached, the cells were replaced in fresh complete medium for 24 hours. Cell culture supernatants were harvested after centrifugation to remove cell debris. Various cell supernatants were diluted accordingly to fit into the range of standard curves. Patient sera were diluted 400 folds prior to the assay to fit into the range of standard curve.

### Western Blot Analysis and ERK and AKT Phosphorylation

Cellular proteins were extracted with 4 × SDS Laemmli sample buffer without the dye. Protein concentrations from all the samples were determined using Bio-Rad D_c_ Protein Assay Reagents. Equal amounts of proteins were analyzed by western blotting as described [37]. Prostate cancer cells and prostate CAF cells were cultured until sub-confluence and switched to serum-free medium (SFM) for 48 and 24 hours, respectively. Purified human TIMP-1 (250ng/ml, R & D Systems) was applied to the serum-starved prostate cancer cells and prostate CAF cells for 3 and 12 hours. The cells were then lysed and equal amounts of extracted proteins were analyzed by western blotting with anti-phospho-ERK1/2 or -phospho-AKT and anti-ERK1/2 or anti-AKT to detect phosphorylated and total amounts of ERK1/2 and AKT proteins, respectively. 

### Cell Proliferation and Migration Assay

Cell proliferation assay was performed by seeding prostate CAFs and prostate cancer cells at 2 × 10^3^ or 4× 10^3^ cells/well as detailed in figure legends into 96-well plates in triplicate in different culture media as detailed in figure legends. After 24 hours, the cells were switched to fresh media in the absence or presence of 250ng/ml of TIMP-1 or the conditioned media. These cells were fed with fresh media with or without TIMP-1 or the conditioned media every day. The cell proliferation assays were performed on a set of the cells every day using the Premix WST1 Reagent (TaKaRa) following the manufacturer’s instruction.

Transwell migration assays were performed as described [37]. Briefly, 0.5×10^6^ or 1×10^6^ cells/ml prostate CAFs in 2% FBS DMEM/F12 media were placed in the upper chambers of Transwell inserts (Costar) in triplicates. 2% FBS-DMEM/F12 media in the presence of 250ng/ml of TIMP-1 or the conditioned media was added in the bottom chambers of each well. After incubation at 37 degrees for 30 hours, the CAFs that migrated through the transwell and reached underside of the inserts were fixed and stained using the Diff-Quick Stain Set (Siemens). The stained cells in 20 randomly selected 200× microscopic fields were then countered using the QCapture Imaging Software. 

### Cancer in vivo Growth Experiments

All animal procedures were performed according to the NIH guidelines and approved by the Institutional Animal Care and Use Committee of the Mount Sinai School of Medicine. Pooled populations of PC3, 22RV1, LAPC-4, HCT116, and HT-29 cells transduced with retroviruses carrying empty expression vectors or overexpressing TIMP-1 were used for subcutaneous cancer growth experiments. Briefly, 2 × 10^6^ (PC3, HCT116, and HT-29) or 5 × 10^6^ (22RV1 and LAPC-4) cells were injected subcutaneously into each immunocompromised Rag-2/II2rg mouse (Taconic). Six mice were used for each type of the infected cancer cells. After solid tumors became visible, longest and shortest diameters of solid tumors were measured using a digital caliper in the intervals and length as indicated in the figures. Tumor volumes were calculated using the following formula: tumor volume=1/2 × (shortest diameter)^2^ × longest diameter (mm^3^). At end of the experiments, all the tumors were fixed and sectioned for histological and immunological analyses.

### Histology and Immunohistochemistry (IHC)

Histology was performed as described [35]. Paraffin sections derived from prostate and colon cancer patient samples (CHTN) and prostate and colon cancer tissues from the *in vivo* experiments were reacted with different antibodies as detailed in figure legends. 

### Statistics

All the data were expressed as mean +/- SD. Student’s *t* test was used to analyze statistical differences between the control and experimental groups. Differences were considered statistically significant at *p*<0.05.

## Results

### Serum TIMP-1 levels are elevated in prostate cancer patients compared to men without cancer

**Figure 1:**
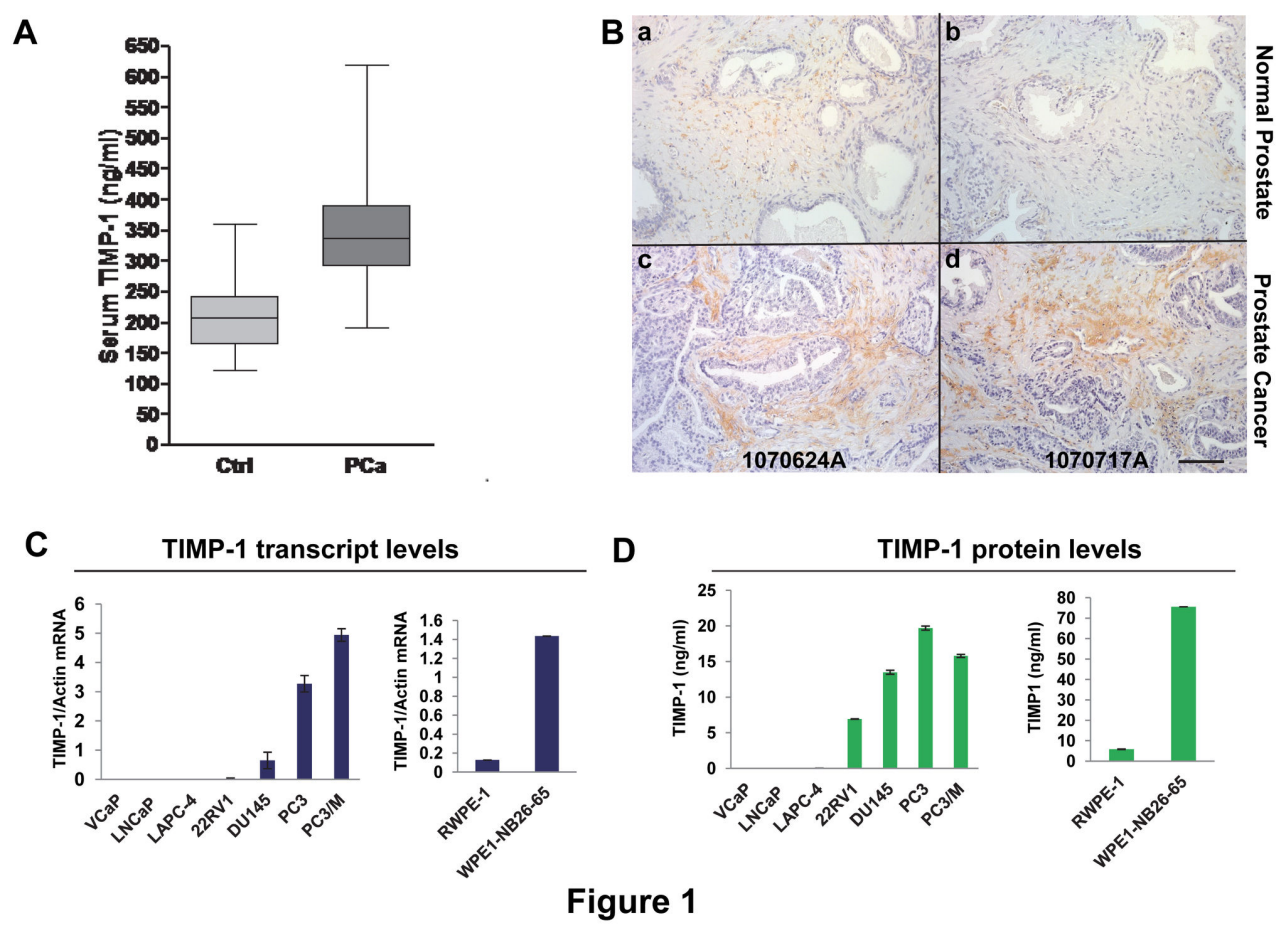
TIMP-1 is up regulated in prostate cancer. **A**. Serum TIMP-1 was significantly elevated in prostate cancer patients (*p*<0.001) as measured by sandwich ELISA (R&D Systems). n=16 for normal individuals (Ctrl) and n=140 for prostate cancer patients (Pca). **B**. Representative images show that TIMP-1 is elevated in prostate cancer stroma (n=6) comparing to their normal counterparts (n=6): TIMP-1 protein levels in human prostate cancer tissues (B-c and d) and normal human prostate tissues (B-a and b) were assessed by immunohistochemistry (IHC) using anti-human TIMP-1 antibody (R & D Systems). 1070624A: prostate adenocarcinoma sample from a 73 year old WM (Gleason score of 4+4=8; PSA of 5.6). 1070717A: prostate adenocarcinoma tissue from a 53 year old WM (Gleason score of 4+3=7; PSA of 8.03). Bar, 100 µm. **C**-**D**. TIMP-1 expression was elevated in more aggressive prostate cancer cell lines. TIMP-1 mRNA (**C**) and protein (**D**) expression in various prostate cancer cell lines were measured by real-time qPCR (**C**) and sandwich ELISA (**D**), respectively. TIMP-1 mRNA was normalized to beta-actin in a duplex qPCR. TIMP-1 protein in conditioned media from the indicated cell lines was measured by TIMP-1 ELISA Duoset (R&D Systems).

Serum TIMP-1 has been reported to be elevated in breast, gastric, colon cancer and several other cancer types. In this study, we compared serum TIMP-1 levels in 140 prostate cancer patients in various disease stages and 16 males without known evidence of prostate cancer including patients with benign prostatic hyperplasia by sandwich ELISA. Our results showed that prostate cancer patients had an average serum TIMP-1 concentration of 351.4 ng/ml, significantly higher than the average concentration of 211.0 ng/ml seen in men without prostate cancer recruited from a urology clinic (*p*<0.001) (Figure 1A). 

### Human prostate cancer stroma express higher levels of TIMP-1

To determine the TIMP-1 expression level in a variety of human cancer types, we searched gene expression datasets at www.oncomine.org, which showed that TIMP-1 transcript levels are up-regulated in most human cancer types including colon cancer comparing to their normal counterparts (Figure S1). In addition to Figure 1A, studies have shown that TIMP-1 levels in plasma of prostate cancer patients are elevated and the elevated TIMP-1 levels predict for decreased survival of castration-resistant (CR) prostate cancer patients [25,38]. To further assess localization of TIMP-1 in human prostate cancers and determine the potential source(s) of the elevated plasma TIMP-1, we performed immunohistochemistry (IHC) on a panel of human prostate cancer samples and normal prostate tissues. The results showed that TIMP-1 protein is up regulated mainly in prostate cancer stroma compared to its normal counterpart (Figure 1B). 

To determine the TIMP-1 expression levels in human prostate cancer cells, we assembled a panel of human prostate cancer cell lines (LNCaP, VCaP, LAPC-4, 22RV1, DU145, PC3 and PC3/M), an immortalized normal prostate epithelial line (RWPE-1) and its malignant derivative (WPE1-NB26-65) [39]. Levels of TIMP-1 mRNA and protein in these cells were assessed, respectively, by qPCR analyses and sandwich ELISA using conditioned media derived from these cell lines. Both experimental results showed that prostate cancer cell lines express various levels of TIMP-1 and that more malignant cell lines such as DU145, PC3 and PC3/M expressed higher levels of TIMP-1 at both mRNA and protein levels (Figure 1C-D). Consistently, WPE1-NB26-65, a malignant cell line derived from immortalized prostate epithelial cell line RWPE-1, expressed approximately 10 times more TIMP-1 than its parental cell line (Figure 1C-D), indicating that increased TIMP-1 expression is associated with prostate cancer progression. Together, these results work against the established dogma and suggest a pro-tumor instead of anti-cancer activity of TIMP-1.

### TIMP-1 promotes prostate cancer growth

**Figure 2:**
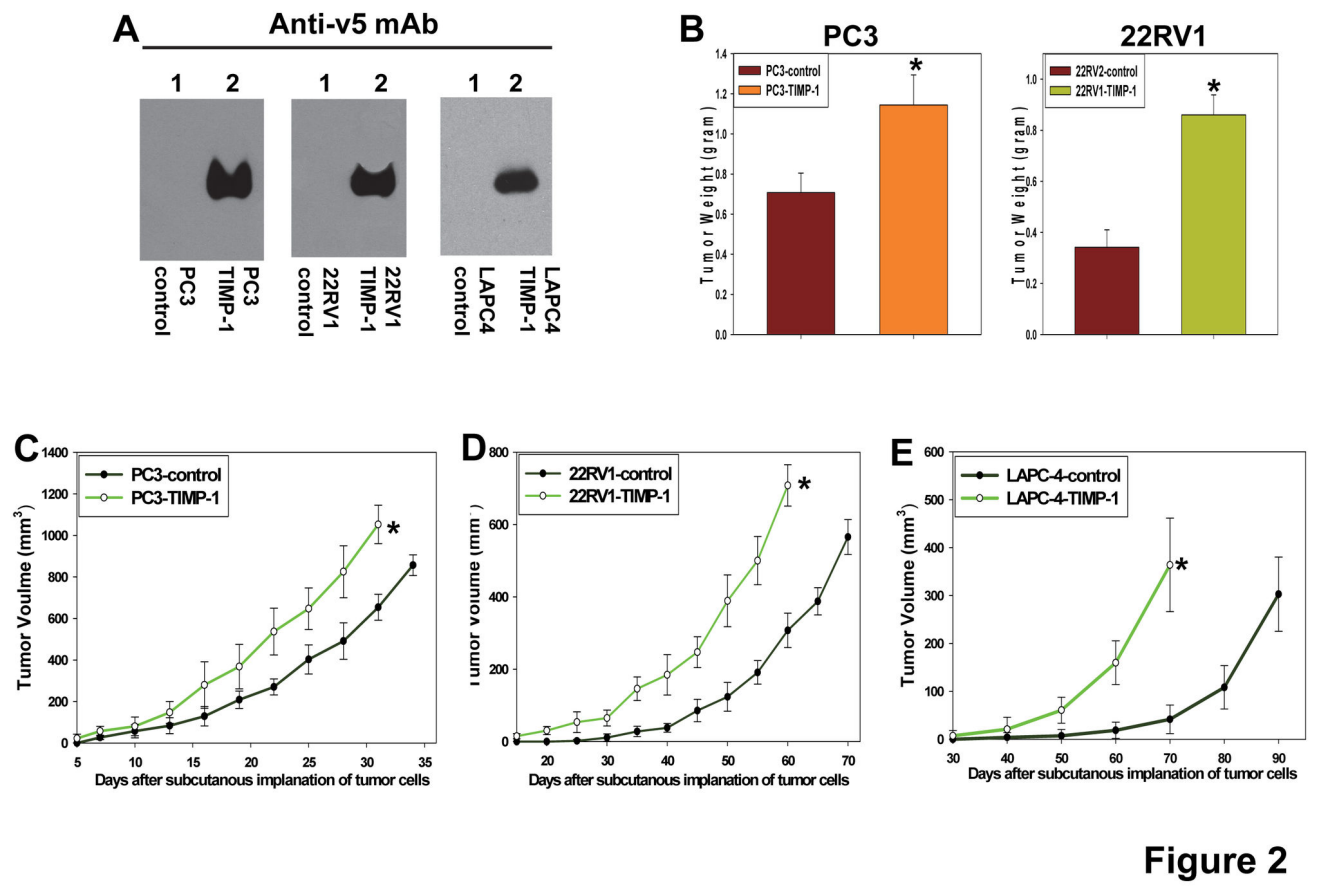
TIMP-1 promotes in vivo growth of prostate cancers. **A**. Establishment of pooled populations of PC3, 22RV1, and LAPC-4 cells expressing v5 epitope tagged TIMP-1 or transduced with the empty expression vector alone (controls). Secreted v5-tagged TIMP-1 was detected by anti-v5 mAb (Invitrogen). **B**. TIMP-1 promotes PC3 and 22RV1 prostate cancer growth *in* *vivo*. Weights of the tumors derived from PC3 and 22RV1 prostate cancer cells expressing TIMP-1v5 or infected with the empty expression constructs (PC3-control and 22RV1-control) were measured 5 or 10-weeks, respectively, after subcutaneous implantation of the cancer cells into Rag-2/II2rg mice (Taconic). n=6. ^*^ *p*<0.05. **C**-**E**. Growth rates of the subcutaneous prostate tumors derived from PC3 (**C**), 22RV1 (**D**), and LAPC-4 (**E**) cells expressing TIMP-1v5 or transduced with the empty expression vector (controls) as indicated in the panels. The growth rates are expressed as the mean of tumor volumes (mm^3^) +/- SD. Six mice were used for each type of transduced prostate cells. ^*^ *p*<0.05.

To determine the potential role of TIMP-1 in prostate cancer progression, we selected three prostate cancer cells, PC3, 22RV1, and LAPC-4, because they are capable of forming tumors in immunocompromised mice, to overexpress v5-epitope tagged exogenous TIMP-1 (Figure 2A). PC3, 22RV1, and LAPC-4 cells transduced with the empty expression vectors were used as the negative controls. The increased expression of v5-epitope tagged TIMP-1 was validated by Western blot analyses of cell culture supernatants of the pooled populations of transduced prostate cancer cells (Figure 2A). These pooled populations of transduced cells were implanted subcutaneously into Rag-2/II2rg immunocompromised mice and tumor growth rates were assessed and calculated as described in materials and methods. Our results showed that increased expression of TIMP-1 significantly promoted growth of PC3, 22RV1, and LAPC-4 prostate cancer cells *in vivo* (Figure 2B-E) and the promotive effect of TIMP-1 is more potent on 22RV1 and LAPC-4 cells, presumably due to the lower endogenous level of TIMP-1 in these cells comparing to PC3 cells. 

### TIMP-1 promotes accumulation of the cancer-associated fibroblasts (CAFs) in vivo

**Figure 3:**
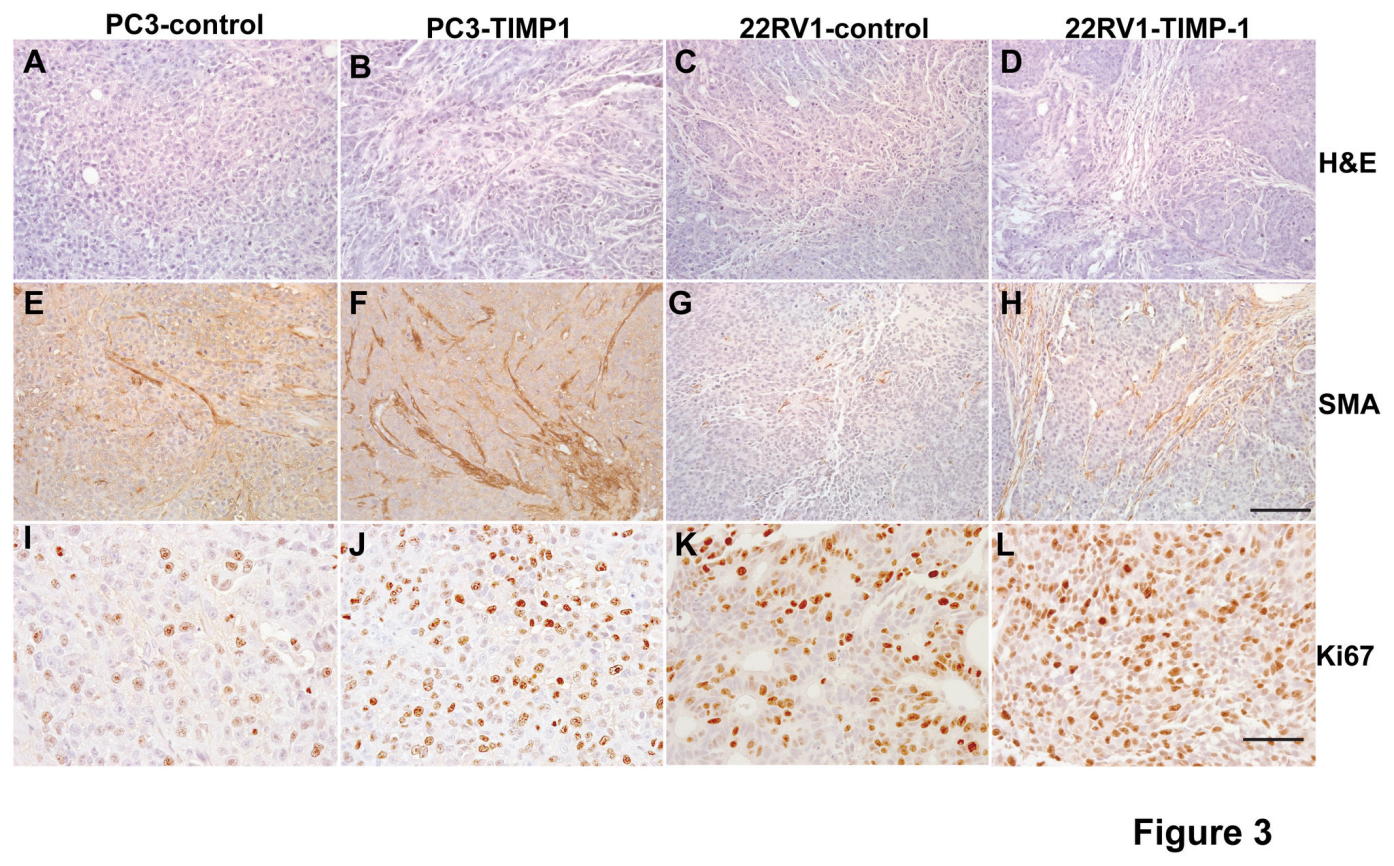
TIMP-1 promotes accumulation of the cancer-associated fibroblasts (CAFs) and promotes prostate cancer proliferation in vivo. The tumor sections were derived from PC3/22RV1-control cells and PC3/22RV1-TIMP-1 cells. The sections were stained with H&E (A-D) to reveal the tumor histology, with anti-alpha smooth muscle actin (anti-SMA, R&D Systems) to detect the cancer associated fibroblasts (CAFs, E-H), and with anti-Ki67 antibody (BD) to highlight the proliferating prostate cancer cells (I-L). Bar, 100 µm in A-H and 50 µm in I-L. .

To gain insights of potential mechanism underlying the pro-growth effect of TIMP-1 in prostate cancer, we performed immunohistochemistry (IHC) analyses of the tumor sections derived from these *in vivo* experiments and observed that the tumor sections derived from the prostate cancer cells with increased expression of TIMP-1 display increased amount of CAFs (Figure 3-top and middle panels). The CAFs were detected using anti-alpha-smooth muscle actin (SMA) antibody, which is routinely used to highlight CAFs [40-42]. It has been well established that CAFs play important roles in promoting cancer progression and mediating therapeutic resistance [43,44] and that CAFs are associated with increased risk of tumor invasion and metastasis [45-47].

### TIMP-1 promotes proliferation of prostate cancer cells in vivo

It is established that CAFs secrete many growth factors that in turn regulate tumor proliferation and survival [43,46,47]. To assess how increased expression of TIMP-1 affects prostate cancer cell proliferation *in vivo*, we performed immunohistochemical analysis of Ki67, a proliferation marker, on the tumor sections. Our results showed that increased expression of TIMP-1 significantly increased the number of Ki67 positive and therefore proliferating prostate cancer cells (Figure 3-bottom panels). 

**Figure 4:**
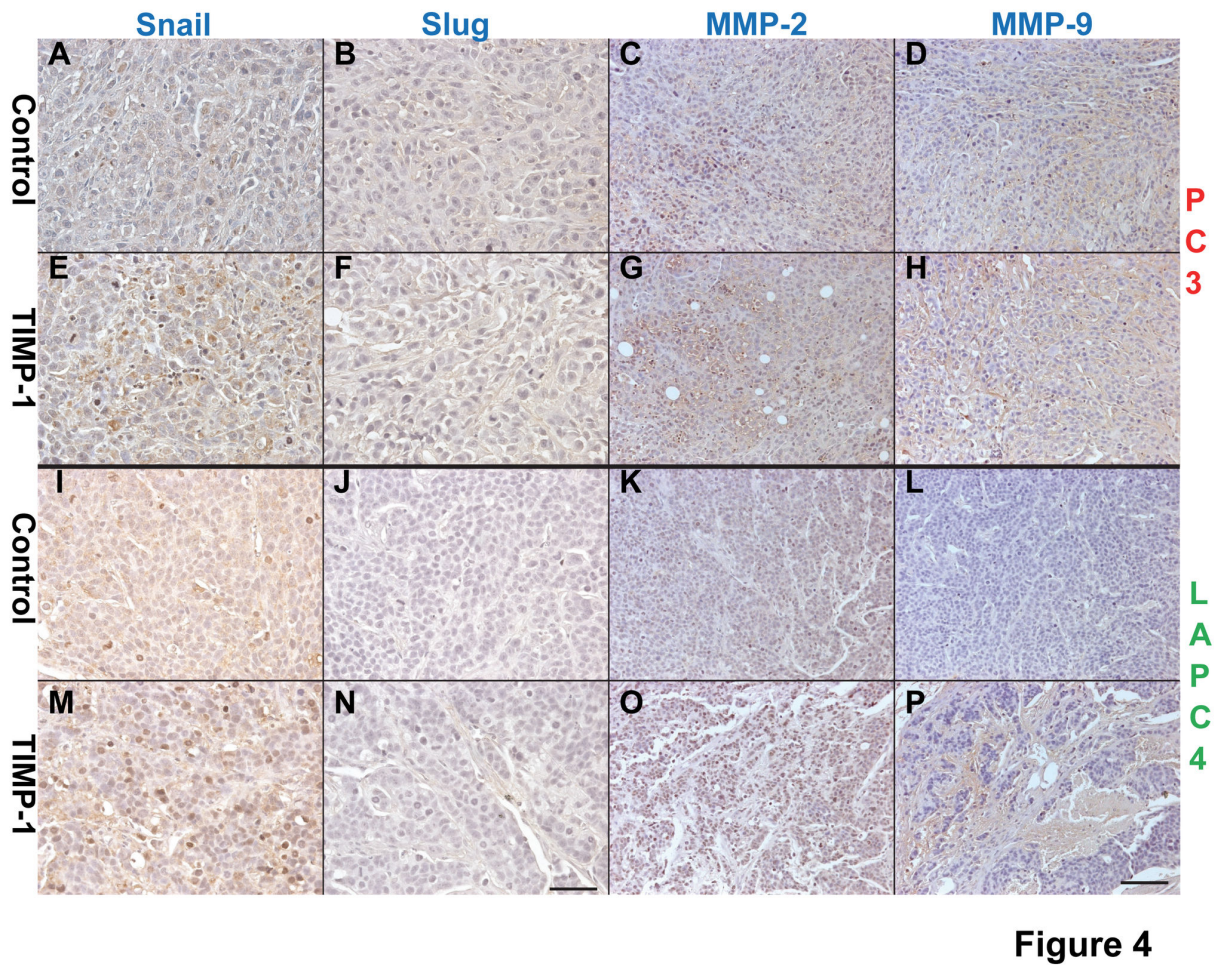
TIMP-1 enhances expression of Snail, MMP-2, and MMP-9 in prostate cancers. Expression levels of several EMT markers, such as Snail (A, E, I, M), Slug (B, F, J, N), MMP-2 (C, G, K, O), and MMP-9 (D, H, L, P) were assessed on the tumor sections derived from PC3/LAPC-4 control cells (A-D and I-L, respectively) and PC3/LAPC-4-TIMP-1 cells (E-H and M-P, respectively). The results show that TIMP-1 enhances expression of Snail, MMP-2, and MMP-9 but not Slug in the prostate cancer tissues. Bar, 50 µm in A, B, E, F, I, J, M, N and 100 µm in C, D, G, H, K, L, O, P.

TIMP-1 is known to play a role in promoting the epithelial-mesenchymal transition (EMT) [48]. To test whether increased expression of TIMP-1 induces expression of the EMT markers, we analyzed the tumor sections derived from the *in vivo* experiments for the expression of Snail, Slug, MMP-2, and MMP-9. Our results showed that expression of Snail, MMP-2 and MMP-9 but not Slug is up-regulated in the prostate cancer sections derived from PC3 and LAPC-4 cells overexpressing TIMP-1 when compared to the sections derived from the control PC3 and LAPC-4 cells (Figure 4). 

TIMP-1 is elevated in the stroma of aggressive and metastatic human colon cancer and increased expression of TIMP-1 promotes colon cancer growth and accumulation of CAFs in colon cancer

**Figure 5:**
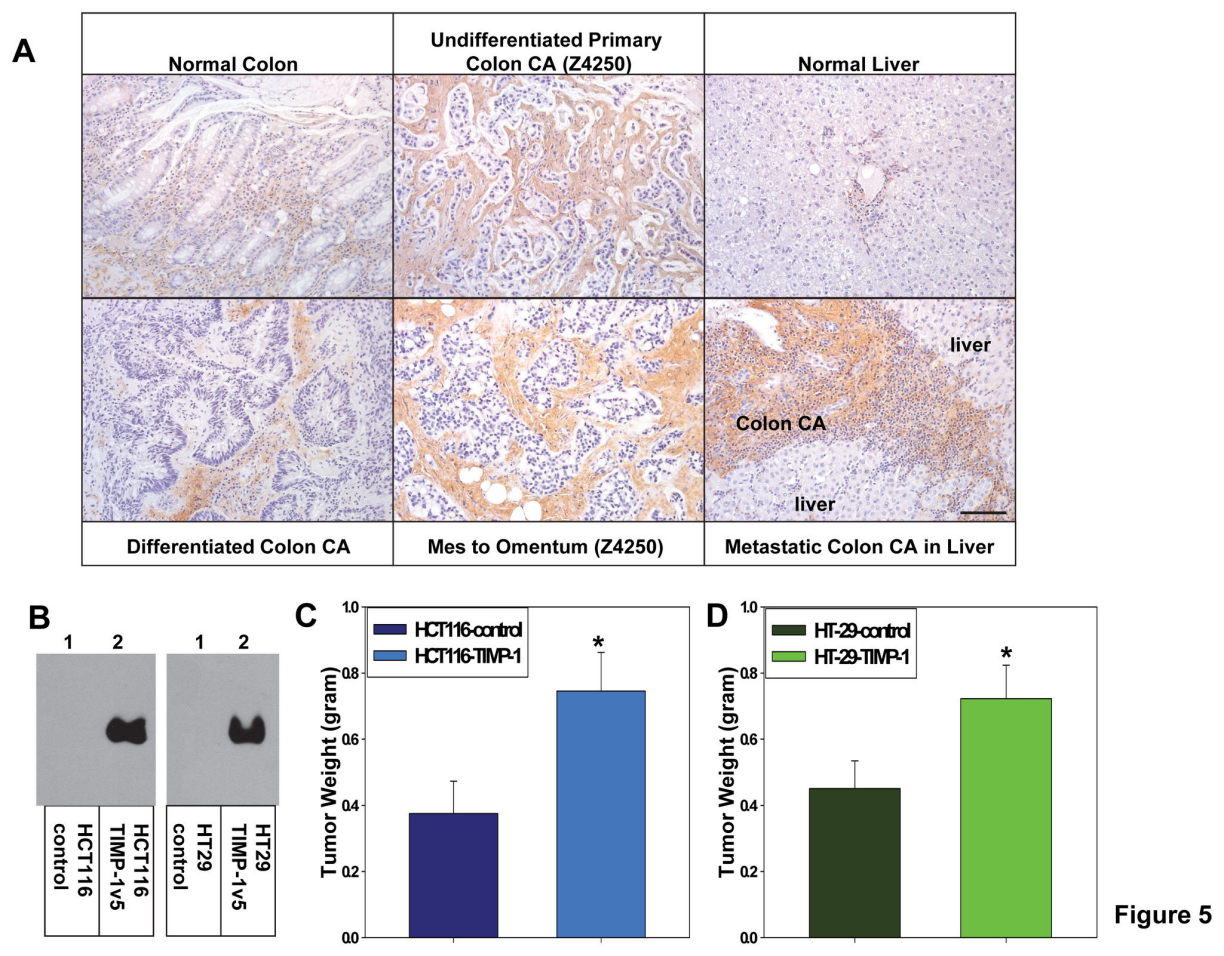
TIMP-1 is elevated in the stroma of more aggressive human colon cancer and TIMP-1 promotes growth of human colon cancers in vivo. **A**. Representative images show that TIMP-1 level is elevated in the stroma of colon cancer (n=6, top middle and bottom panels) comparing to that of normal colon (n=6, top left panel). Stroma of undifferentiated/higher grade colon cancer (n=6, the top middle panel, Z4250) and metastatic colon cancers (n=6, bottom middle and bottom right panels, Z4250 and 4081525) displayed higher levels of the stroma associated TIMP-1 comparing to that of differentiated/lower grade colon cancer (bottom left, Z4265), primary colon cancer (top middle panel), or the normal counterparts (normal colon, top left; normal liver, top right panel). Bar, 100 µm. **B**. Western blot analyses using anti-v5 antibody (Invitrogen) were performed to assess expression of exogenous v5-epitope tagged TIMP-1 (TIMP-1v5) in HCT116 (left panel) and HT-29 (right panel) human colon cancer cells. **C**-**D**. TIMP-1 promotes HCT116 and HT-29 colon cancer growth *in* *vivo*. Weights of the tumors derived from HCT116 and HT-29 colon cancer cells expressing TIMP-1v5 or infected with the empty expression constructs (controls) were measured 6 weeks after the subcutaneous implantation of the cancer cells. n=6. ^*^ *p*<0.05.

To further validate the general pro-tumor activity of TIMP-1 and its general effect on CAFs, we assess TIMP-1 levels in colon cancer and normal colon samples. We found that TIMP-1 level is elevated in the stroma of colon cancer comparing to that of normal colon and that stroma of undifferentiated/higher grade colon cancer and metastatic colon cancers displayed higher levels of TIMP-1 comparing to stroma of differentiated/lower grade colon cancer (Figure 5A). We then assessed the effect of TIMP-1 on colon cancer growth *in vivo*. Our results showed that increased expression of TIMP-1 promotes *in vivo* growth of human HCT116 and HT-29 colon cancer cells (Figure 5 B-D). Taken together, these results indicated that TIMP-1 not only serves as a prognostic marker for multiple cancer progression but also promotes the cancer progression.

To confirm the effect of TIMP-1 on CAFs, we performed similar IHC analyses on the tumor sections derived from the *in vivo* studies of HCT116 colon cancers with or without increased expression of TIMP-1. We found that comparing to tumors derived from HCT116-control cells (Figure S2A-C), increased expression of TIMP-1 (HCT116-TIMP-1) promotes accumulation of colon CAFs within the tumors (Figure S2D-F). Together, these results provide first support for a novel role of TIMP-1 in regulating the CAF infiltration and/or expansion. 

### TIMP-1 promotes prostate CAF cell proliferation and migration and activates Erk1/2 kinases

**Figure 6:**
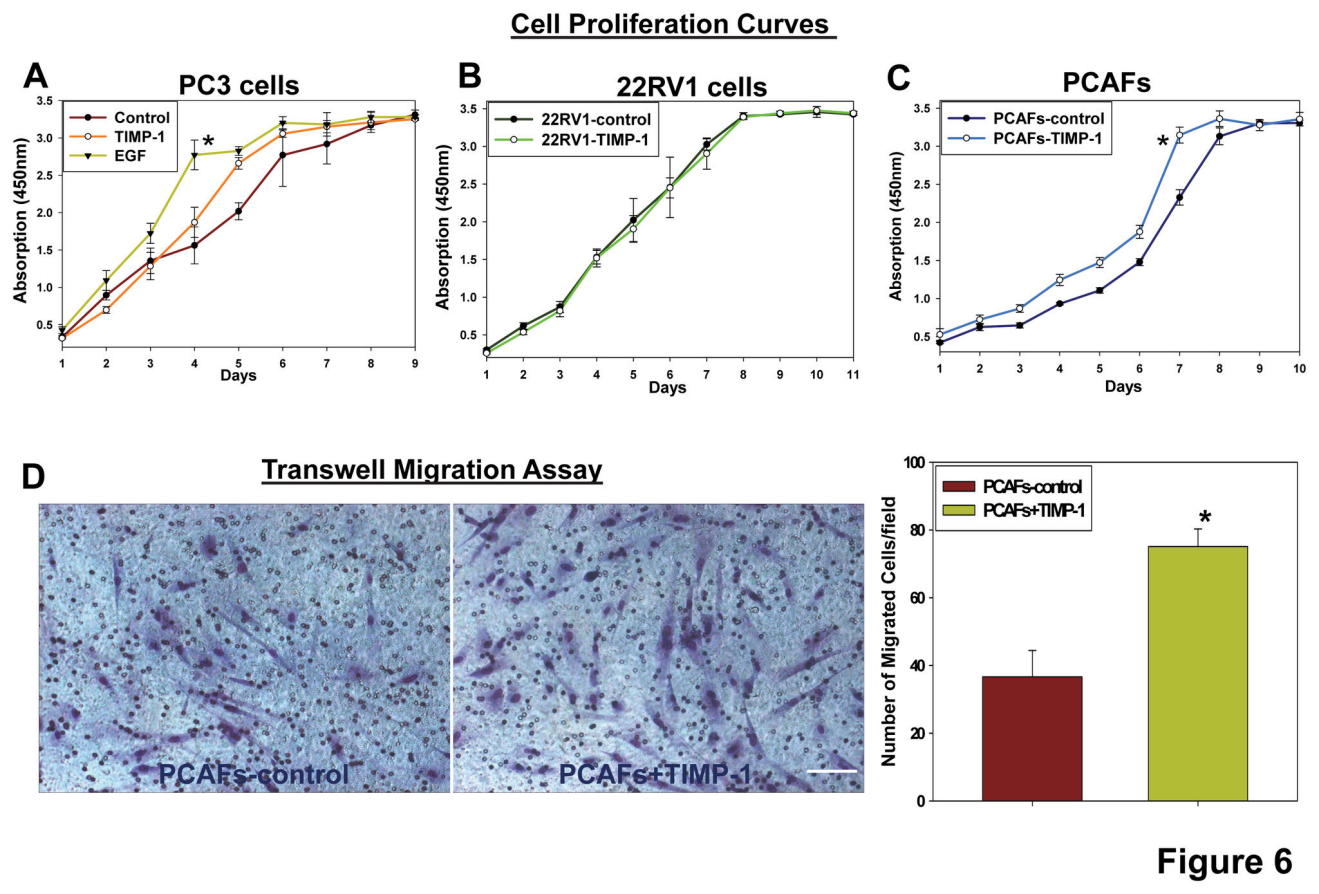
TIMP-1 promotes the prostate CAF proliferation and migration through transwells. **A**-**C**. Prostate CAFs or prostate cancer cells were seeded at 2 x 10^3^ cells/well into 96-well plates in triplicate. Prostate cancer cell and prostate CAF proliferation assays were performed every day using a set of 96-well plates using Premix WST1 kit (TaKaRa) following the manufacturer’s instruction. ^*^ *p*<0.05. **D**. Prostate CAFs were assessed for their motility across transwell barrier over the course of 30 hours in the presence or absence of 250ng/ml of TIMP-1. 0.5 x 10^6^ cells/ml prostate CAFs were placed in the upper chambers of Transwell inserts (Costar) in triplicates. Representative images of prostate CAF cells migrated through the transwell inserts are shown. The prostate CAFs migrated through transwell inserts in 20 random selected 200 x microscopic fields were counted. ^*^ *p*<0.05. A-D, the results show representative means +/-SDs of triplicates of one of two independent experiments.

To reveal the cellular mechanism by which TIMP-1 promotes CAF accumulation and cancer progression *in vivo*, we first assessed the effects of the conditioned media (CM) derived from 22RV1-control and 22RV1-TIMP-1 cells on prostate CAF proliferation and migration. We found that the CM derived from 22RV1-TIMP-1 cells more effectively promoted prostate cancer proliferation and transwell migration when compared to the CM derived from 22RV1-control cells (Figure S3). To further determine whether purified TIMP-1 can exert similar effects on prostate CAFs, we performed additional experiments using purified human TIMP-1. We found that TIMP-1 significantly promoted proliferation of prostate CAF but not that of prostate cancer cells (Figure 6 A-C) and that TIMP-1 significantly enhanced motility of these CAFs (Figure 6D), suggesting that the TIMP-1-mediated accumulation of prostate CAFs is likely resulted from both enhanced infiltration and expansion of prostate CAFs within the tumors. 

**Figure 7:**
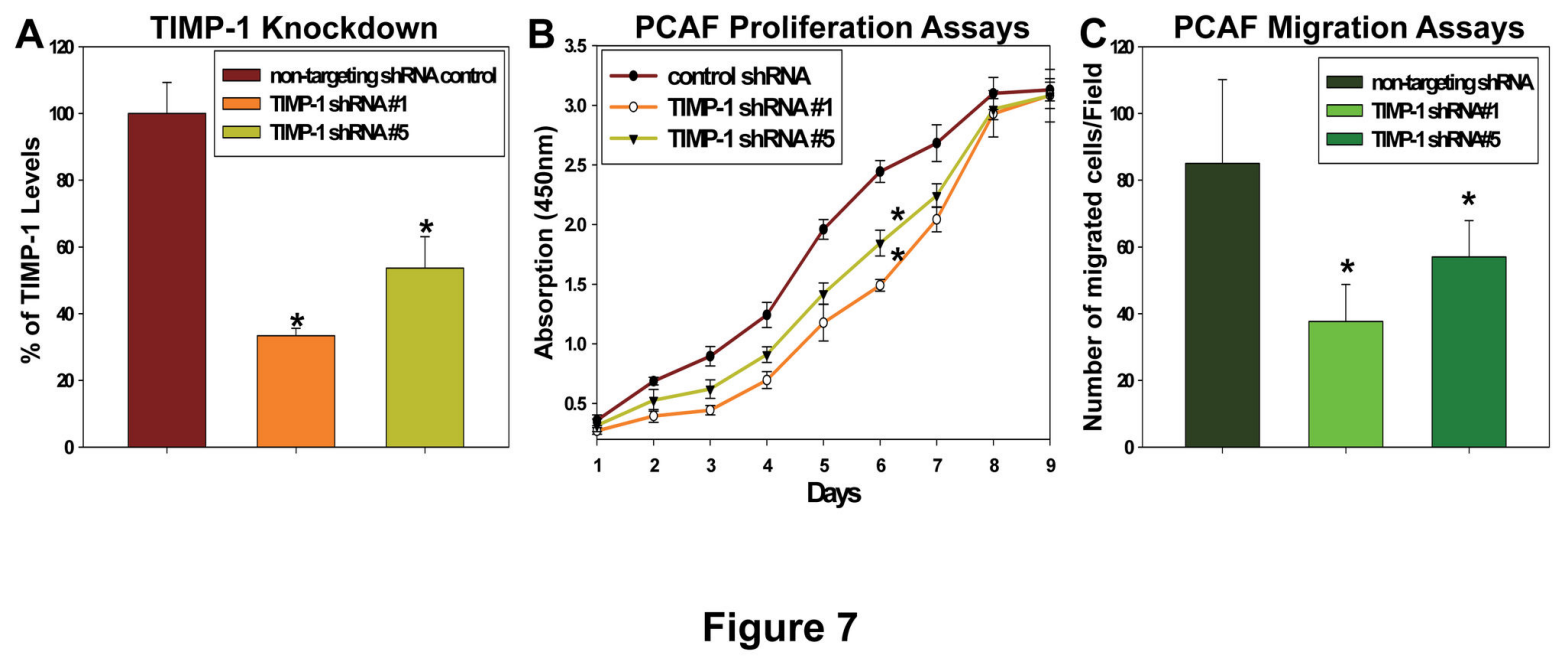
TIMP-1 knockdown inhibits prostate CAF proliferation and migration through transwells. **A**. Relative levels of TIMP-1 in prostate CAFs infected with non-targeting shRNAs or shRNAs against TIMP-1 were assessed by ELISA and the results showed that two TIMP-1 shRNAs knocked down TIMP-1 expression by ~70% and ~50%, respectively. **B**. Prostate CAFs were seeded at 4 x 10^3^ cells/well into 96-well plates in triplicate and prostate CAF proliferation assays were performed every day using a set of 96-well plates using Premix WST1 kit (TaKaRa). ^*^ *p*<0.05. **C**. Prostate CAFs were assessed for their motility across transwell barrier over the course of 30 hours in the presence or absence of 250ng/ml of TIMP-1. 1 x 10^6^ cells/ml prostate CAFs were placed in the upper chambers of Transwell inserts (Costar) in triplicates. The prostate CAFs migrated through transwell in 20 random selected 200 x microscopic fields were counted. ^*^ *p*<0.05.

**Figure 8:**
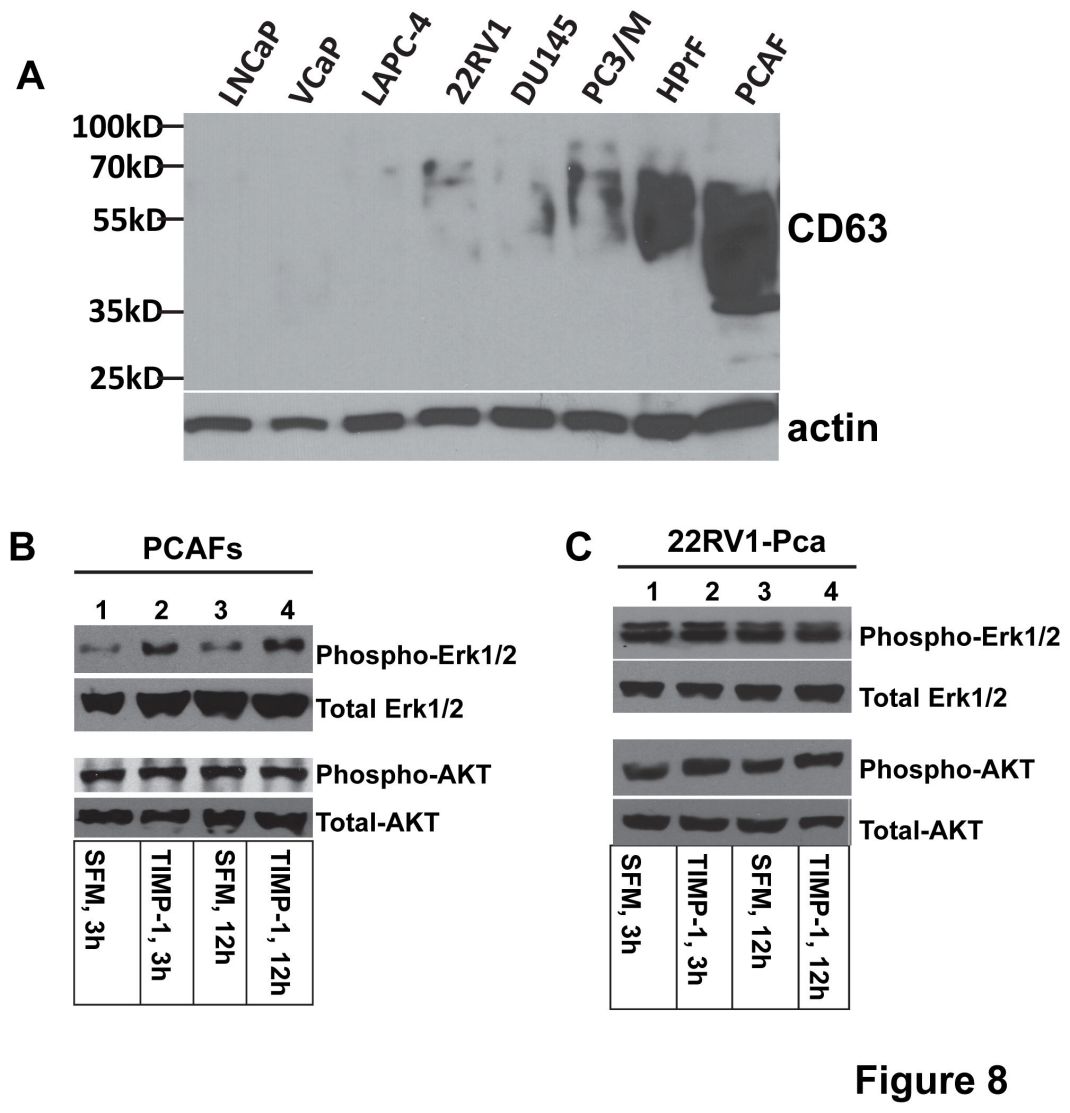
Prostate CAFs express a higher level of TIMP-1 receptor, CD63, and TIMP-1 promotes activation of ERK1/2 kinases in prostate CAFs. **A**. Expression of CD63 protein in various human prostate cancer cell lines, human prostate fibroblasts (HPrF), and human prostate CAFs (PCAFs) was determined by western blotting using anti-CD63 antibody, which recognizes heterogeneous glycosylated CD63 protein. Actin was used as a control for protein loading (lower panel). **B**-**C**. Serum-starved prostate CAF cells (**B**) and 22RV1 prostate cancer cells (**C**) were supplied with serum free medium (SFM) or SFM containing 250ng/ml of TIMP-1 for 3 and 12 hours. The cells were lysed and proteins were analyzed by Western blotting using anti-phospho-ERK1/2 or anti-phospho-AKT antibodies (upper panels), or anti-ERK1/2 or anti-AKT antibodies (bottom panels).

**Figure 9:**
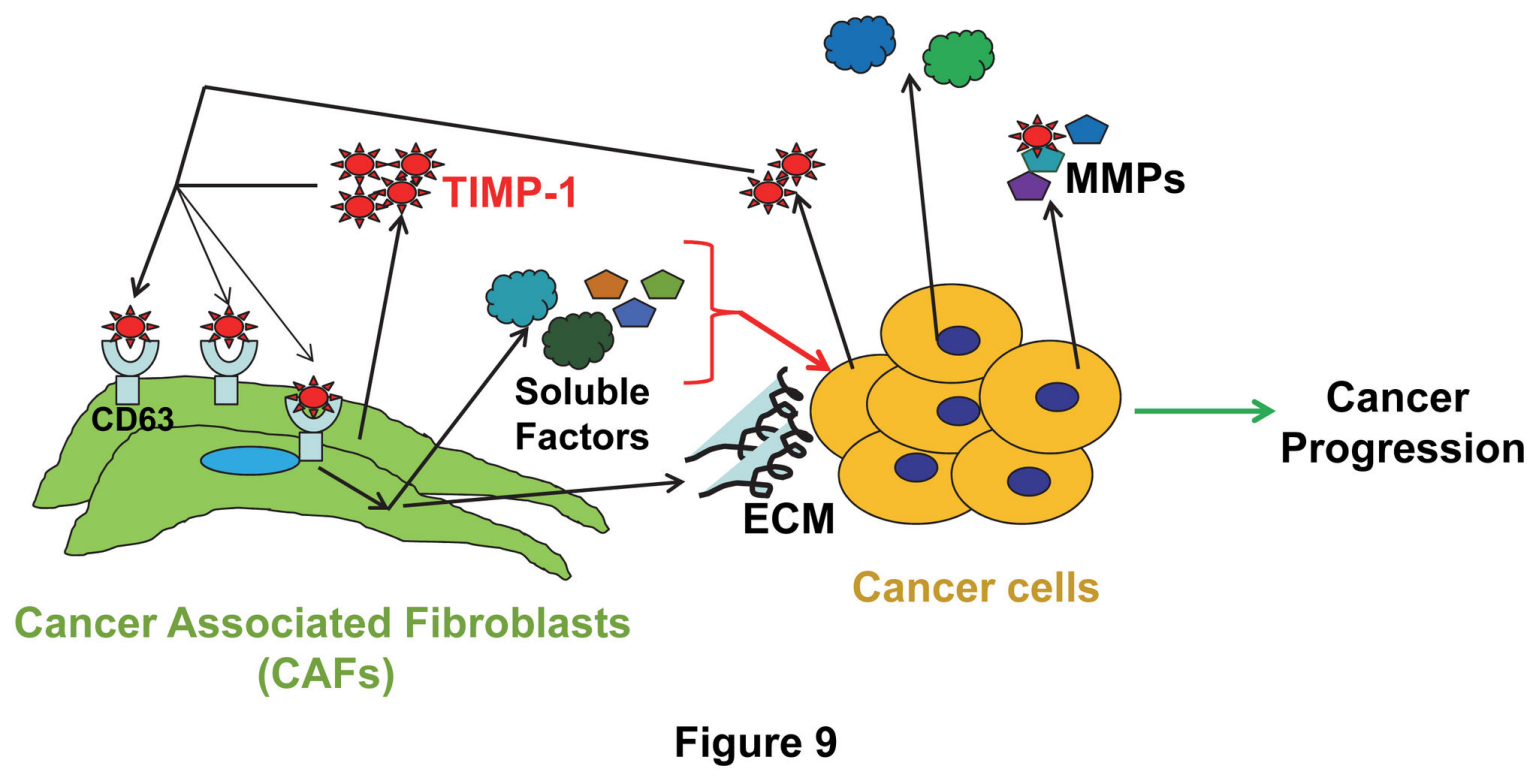
*A model* of action of TIMP-1 during tumor progression : Increased expression of TIMP-1 by tumor cells and CAFs leads to increased CAF proliferation and migration through binding of TIMP-1 to the TIMP-1 receptor, CD63 expressed on CAFs, which leads to accumulation of CAFs within cancer tissues. These CAFs in turn provide a pro-tumor microenvironment to facilitate the cancer progression by secreting pro-tumor growth factors/cytokines, modulating tumor angiogenesis, and regulating infiltration and expansion of inflammatory cells.

To further confirm the TIMP-1 effects on prostate CAFs, we knocked down TIMP-1 expression in prostate CAFs (Figure 7A) using two different shRNAs (Open Biosystems). We found that TIMP-1 knockdown inhibits prostate CAF proliferation and migration significantly (Figure 7B-C), suggesting that TIMP-1 plays an important role in regulating CAF behaviors, which in turn modulating cancer progression. This notion was further supported by our finding that prostate CAFs but not prostate cancer cells express a higher level of the TIMP-1 receptor, tetraspanin CD63. CD63 is a glycosylated membrane protein displaying heterogeneous molecular weight (Figure 8A). TIMP-1 was found to activate the pro-survival signaling by binding to the CD63/integrin beta1 complex in breast epithelial cells [49,50]. We found that TIMP-1 enhances ERK1/2 activity of serum starved prostate CAFs but not prostate cancer cells (Figure 8B-C), suggesting that the *in vivo* effects of TIMP-1 on prostate cancer cells might be exerted through affecting prostate CAFs indirectly (Figure 9).

## Discussion

### TIMP-1 serves as a novel cancer therapeutic target

Results from several recent clinical studies have demonstrated that TIMP-1 levels in cancer patient plasma are elevated and the elevated TIMP-1 levels are associated with worse clinical outcomes in many cancer types including prostate and colon cancer [23-34]. A study also showed that elevated TIMP-1 in colorectal cancer correlates with lymph node and distant metastases [23]. However, it is still unknown whether TIMP-1 serves merely as a biomarker of cancer progression or functions to promote cancer progression. In this study, we establish that increased expression of TIMP-1 promotes *in vivo* growth of prostate cancer and colon cancer (Figure 2 and Figure 5). In addition, we showed that TIMP-1 is up-regulated in the cancer stroma compared to their normal counterparts (Figure 1B and Figure 5A). These results established the potential of TIMP-1 as a novel cancer therapeutic target and provide support for development of inhibitors of TIMP-1 as anti-cancer agents. Successful development of TIMP-1 inhibitors that are capable of blocking its pro-tumor activity requires additional future studies to better understand the molecular bases of the pro-tumor activity of TIMP-1 as TIMP-1 displays both MMP-dependent and MMP-independent functions. Different strategies are likely required to block these different activities. 

### The MMP-dependent or MMP-independent activity of TIMP-1

In addition to serving as a naturally occurring inhibitor of MMPs, TIMP-1 also displays several MMP-independent effects, including promoting tumor proliferation [51] and angiogenesis [52], and inhibiting apoptosis [53]. We showed that TIMP-1 promotes progression of prostate and colon cancer (Figure 2 and Figure 5). More detailed future studies are required to determine whether the pro-tumor effect of TIMP-1 is MMP-dependent or MMP-independent. If the former is true, the pro-tumor activity of TIMP-1 could be mediated through inhibition of activities of a subgroup of MMPs that may exert anti-tumor activity during late stages of tumor progression, which notion could offer an underlying reason for the failed clinical trials of the broad spectrum MMPIs that indiscriminately inhibit all MMP activities. If the pro-tumor activity of TIMP-1 is MMP-independent and exerted through its indirect promotive effect on CAFs and the TIMP-1 receptor, CD63 expressed by CAFs. CD63 may then serve as an important therapeutic target against the cancer types with elevated stroma TIMP-1 levels. Our results showed that prostate CAFs but not prostate cancer cells express a high level of CD63 and that prostate CAFs but not prostate cancer cells responded to TIMP-1 of or knockdown of TIMP-1 (Figures 6-7), suggesting that through CD63 receptor expressed by prostate CAFs, TIMP-1 can promote CAF proliferation and migration, which lead to accumulation of CAFs within cancer tissues. Increased CAFs can in turn provide a favorable tumor microenvironment that promotes the cancer progression (Figure 9). 

### The novel effect of TIMP-1 on the cancer associate fibroblasts (CAFs)

It is well established that CAFs promote tumor cell proliferation and invasion, and enhance tumor angiogenesis by secreting many pro-tumor growth/angiogenic factors and cytokines [42,43,46,54-56]. CAFs also promote pro-tumor inflammation through activation of the NF-kappaB signaling pathway [42]. However, less is known about the factors that regulate CAFs. Here, we showed that cancer stroma expresses higher levels of TIMP-1 compared to their normal counterparts (Figure 1B and Figure 5A) and that increased expression of TIMP-1 promotes accumulation of CAFs within prostate/colon cancer tissues (Figure 3 and Figure S2). We also established that TIMP-1 enhances whereas knockdown of TIMP-1 inhibits prostate CAF proliferation and migration and that TIMP-1 promotes activation of ERK1/2 kinase in these CAFs (Figures 6-7 and Figure 8B) but not in prostate cancer cells (Figure 8C) *in vitro*, suggesting that the pro-tumor effect of TIMP-1 is indirectly exerted through the cancer associated fibroblasts, which in turn provides a pro-tumor microenvironment to facilitate the cancer progression (Figure 9). 

Accumulation of CAFs induced by TIMP-1 within prostate and colon cancers can be achieved through stimulating CAF infiltration, expansion/proliferation, and/or trans-differentiation of the resident fibroblasts to CAFs. Even though more detailed studies are required to definitively distinguish these possibilities, the promotive effects of TIMP-1 on prostate CAF proliferation and migration suggest that TIMP-1 promotes infiltration and as well as expansion of CAFs within the tumors. The novel role of TIMP-1 in promoting the infiltration/expansion of CAFs also suggests that TIMP-1 inhibitors developed in the future may have potentials to be used to block the CAF functions and therefore achieving their anti-cancer effects through attacking the tumor microenvironment. 

## Supporting Information

Figure S1 **TIMP-1 transcripts are up-regulated (red color) in different cancer types comparing to their normal counterparts (results were derived from the databases at www.oncomine.org).**
(TIF)

Figure S2 **TIMP-1 promotes accumulation of the cancer-associated fibroblasts in colon cancers.** The tumor sections were derived from HCT116-control cells (**A**-**C**) and HCT116-TIMP-1 cells (**D**-**F**). The sections were stained with H&E (A-B and D-E) to reveal the tumor histology and with anti-alpha smooth muscle actin antibody (anti-SMA, R&D Systems) to detect the CAFs (C and F). Bar, in A, C, D, and F, 100µm and B and E, 50µm.
(TIF)

Figure S3 **The conditioned media (CM) derived from 22RV1-TIMP-1 cells promotes prostate CAF proliferation and migration.** **A**. Prostate CAFs were seeded at 4 x 10^3^ cells/well into 96-well plates in triplicate and supplied with fresh 1:1 mixture of FM and the CM derived from 22RV1-TIMP-1 cells or 22RV1-control cells every day. Prostate CAF proliferation assays were performed every day using a set of 96-well plates using Premix WST1 kit (TaKaRa). ^*^ *p*<0.05. **B**. Prostate CAFs were used to assess their motility across transwell barrier over the course of 30 hours in the presence or absence of the CM derived from 22RV1-TIMP-1 cells or 22RV1-control cells. 1x10^6^ cells/ml prostate CAFs were placed in the upper chambers of Transwell inserts (Costar) in triplicates. 1:1 mixture of FM and the CM derived from 22RV1-TIMP-1 cells or 22RV1-control cells was applied to the bottom chambers of the transwells. The prostate CAFs migrated through transwells in 20 random selected 200x microscopic fields were counted. ^*^ *p*<0.05.
(TIF)
