# Supplementary figures and images for: TIMP-1 Promotes Accumulation of Cancer Associated Fibroblasts and Cancer Progression
Source: PLoS One. 2013 Oct 15;8(10):e77366. doi: 10.1371/journal.pone.0077366 (PMC3797040; doi:10.1371/journal.pone.0077366)

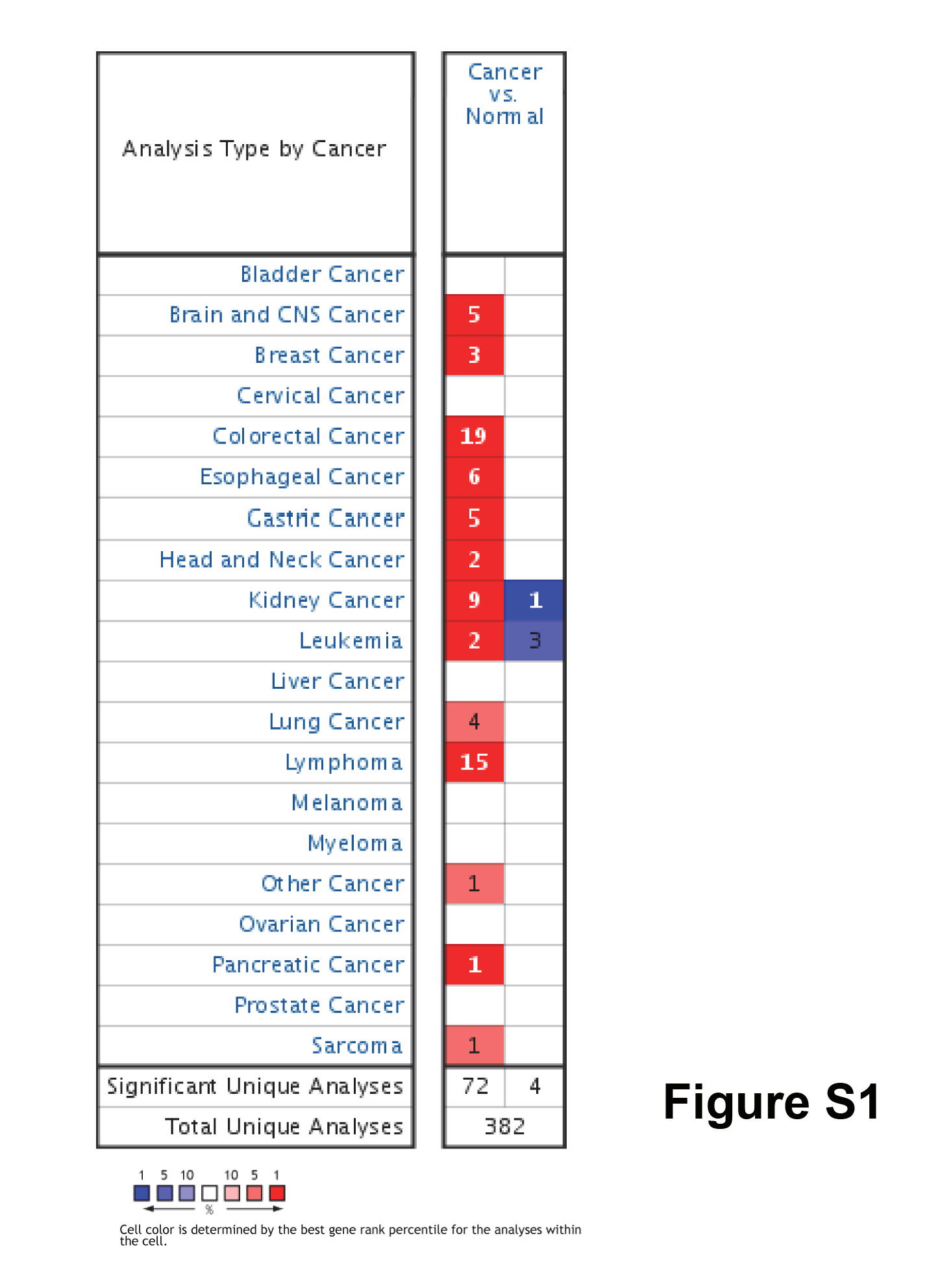

Supplement: Figure S1 — TIMP-1 transcripts are up-regulated (red color) in different cancer types comparing to their normal counterparts (results were derived from the databases at www.oncomine.org). (TIF) [file pone.0077366.s001.tif]

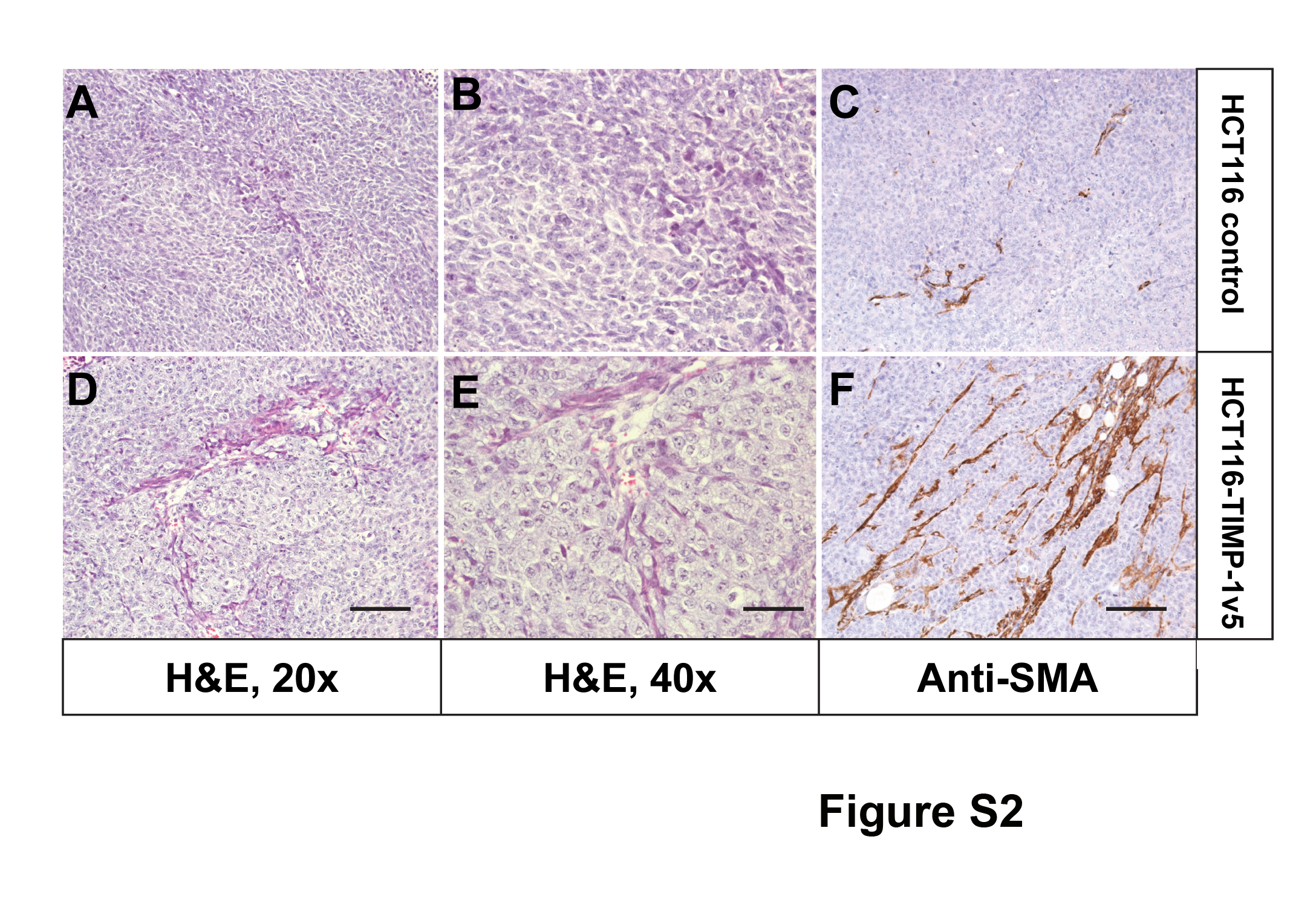

Supplement: Figure S2 — TIMP-1 promotes accumulation of the cancer-associated fibroblasts in colon cancers. The tumor sections were derived from HCT116-control cells (A-C) and HCT116-TIMP-1 cells (D-F). The sections were stained with H&E (A-B and D-E) to reveal the tumor histology and with anti-alpha smooth muscle actin antibody (anti-SMA, R&D Systems) to detect the CAFs (C and F). Bar, in A, C, D, and F, 100µm and B and E, 50µm. (TIF) [file pone.0077366.s002.tif]

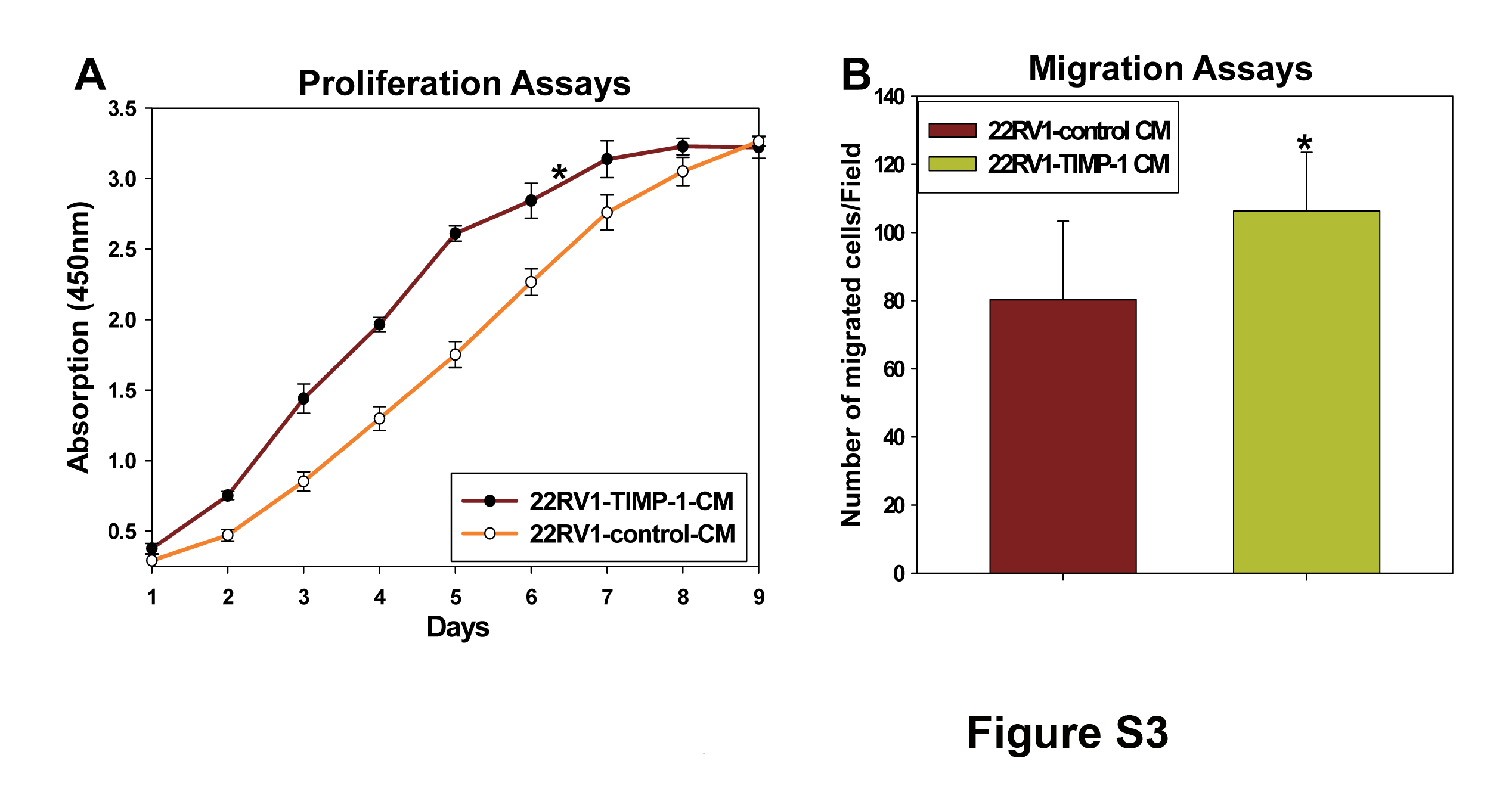

Supplement: Figure S3 — The conditioned media (CM) derived from 22RV1-TIMP-1 cells promotes prostate CAF proliferation and migration. A. Prostate CAFs were seeded at 4 x 10^3^ cells/well into 96-well plates in triplicate and supplied with fresh 1:1 mixture of FM and the CM derived from 22RV1-TIMP-1 cells or 22RV1-control cells every day. Prostate CAF proliferation assays were performed every day using a set of 96-well plates using Premix WST1 kit (TaKaRa). * p<0.05. B. Prostate CAFs were used to assess their motility across transwell barrier over the course of 30 hours in the presence or absence of the CM derived from 22RV1-TIMP-1 cells or 22RV1-control cells. 1x10^6^ cells/ml prostate CAFs were placed in the upper chambers of Transwell inserts (Costar) in triplicates. 1:1 mixture of FM and the CM derived from 22RV1-TIMP-1 cells or 22RV1-control cells was applied to the bottom chambers of the transwells. The prostate CAFs migrated through transwells in 20 random selected 200x microscopic fields were counted. * p<0.05. (TIF) [file pone.0077366.s003.tif]
